# The Accuracy of Clinical Staging of Stage I-IIIa Non-Small Cell Lung Cancer: An Analysis Based on Individual Participant Data

**DOI:** 10.1016/j.chest.2018.10.020

**Published:** 2018-10-26

**Authors:** Neal Navani, David J. Fisher, Jayne F. Tierney, Richard J. Stephens, Sarah Burdett, NSCLC Meta-analysis Collaborative Group, Sarah Burdett, Larysa H.M. Rydzewska, Jayne F. Tierney, Anne Auperin, Thierry Le Chevalier, Cécile Le Pechoux, Jean-Pierre Pignon, Rodrigo Arriagada, David H. Johnson, Jan van Meerbeeck, Mahesh K.B. Parmar, Richard J. Stephens, Lesley A. Stewart, Paul A. Bunn, Bertrand Dautzenberg, David Gilligan, Harry Groen, Aija Knuuttila, Eric Vallieres, Rafael Rosell, Jack Roth, Giorgio Scagliotti, Masahiro Tsuboi, David Waller, Virginie Westeel, Yi-Long Wu, Xue-Ning Yang

**Affiliations:** a Lungs for Living Research Centre, UCL Respiratory and Department of Thoracic Medicine, University College London Hospital, London, England; b MRC Clinical Trials Unit at UCL, London, England

**Keywords:** meta-analysis, non-small cell lung cancer, staging, IPD, individual participant data, NSCLC, non-small cell lung cancer, PET-CT, positron emission tomography-computed tomography, RCT, randomized controlled trial, SABR, stereotactic body radiotherapy

## Abstract

**Background:**

Clinical staging of non-small cell lung cancer (NSCLC) helps determine the prognosis and treatment of patients; few data exist on the accuracy of clinical staging and the impact on treatment and survival of patients. We assessed whether participant or trial characteristics were associated with clinical staging accuracy as well as impact on survival.

**Methods:**

We used individual participant data from randomized controlled trials (RCTs), supplied for a meta-analysis of preoperative chemotherapy (± radiotherapy) vs surgery alone (± radiotherapy) in NSCLC. We assessed agreement between clinical TNM (cTNM) stage at randomization and pathologic TNM (pTNM) stage, for participants in the control group.

**Results:**

Results are based on 698 patients who received surgery alone (± radiotherapy) with data for cTNM and pTNM stage. Forty-six percent of cases were cTNM stage I, 23% were cTNM stage II, and 31% were cTNM stage IIIa. cTNM stage disagreed with pTNM stage in 48% of cases, with 34% clinically understaged and 14% clinically overstaged. Agreement was not associated with age (*P* = .12), sex (*P* = .62), histology (*P* = .82), staging method (*P* = .32), or year of randomization (*P* = .98). Poorer survival in understaged patients was explained by the underlying pTNM stage. Clinical staging failed to detect T4 disease in 10% of cases and misclassified nodal disease in 38%.

**Conclusions:**

This study demonstrates suboptimal agreement between clinical and pathologic staging. Discrepancies between clinical and pathologic T and N staging could have led to different treatment decisions in 10% and 38% of cases, respectively. There is therefore a need for further research into improving staging accuracy for patients with stage I-IIIa NSCLC.

FOR EDITORIAL COMMENT, SEE PAGE 456

The clinical staging of non-small cell lung cancer (NSCLC) is of paramount importance in determining a patient’s prognosis, guiding treatment decisions, and defining clinical trial eligibility, as well as allowing comparison between clinical trials. Incorrect staging of NSCLC may result in inaccurate prognostic information for patients and errors in patient treatment. After extrathoracic metastases have been excluded, tumor and nodal staging are critical in making treatment decisions, as patients with N0 and N1 involvement are generally candidates for surgery. Patients with ipsilateral mediastinal disease (N2) are a heterogeneous group and may be offered chemoradiation therapy or surgery (with preoperative or postoperative chemotherapy). Patients with contralateral (N3) mediastinal (or supraclavicular) nodal disease are offered chemoradiation therapy or palliative treatment options. Therefore, clinical understaging, that is, staging that misses mediastinal metastases or mediastinal invasion of the primary lesion, may risk the patient undergoing radical treatment of the primary lesion for no benefit. Conversely, incorrect clinical overstaging of mediastinal disease may result in surgery being denied to an otherwise operable patient. The current guidance from the Union for International Cancer Control^1^ suggests that when there is doubt about stage, the less advanced, or lower category should be chosen.

The emergence of techniques such as stereotactic body radiotherapy^2^ (SABR) and radiofrequency ablation^3^ to treat early-stage NSCLC in medically inoperable patients has further highlighted the importance of accurate clinical staging. Applying local nonsurgical treatments without the benefit of systematic lymph node dissection runs the risk of being futile if there is clinical understaging with unrecognized mediastinal or systemic disease.

Although the importance of accurate clinical staging is clear and the performance characteristics of individual tests in lung cancer staging are known, fewer data exist on the accuracy of clinical staging of NSCLC and how this relates to the staging techniques employed. Three studies that have been reported all show high levels of inaccurate clinical staging; however, none have demonstrated the impact of erroneous staging on clinical outcome. A prospective study of 383 patients with potentially resectable NSCLC demonstrated that clinically unsuspected N2 disease was found in 14% of patients. Despite routine use of positron emission tomography-computed tomography (PET-CT) scanning,^4^ a post-hoc analysis of 67 patients from the control arm of the Medical Research Council LU22^5^ trial of preoperative chemotherapy suggested that nodal staging was inaccurate in 25% (95% CI, 15%-36%) of patients who underwent PET-CT scanning and mediastinoscopy.^6^ A study comparing clinical and pathologic TNM data, collected for 2,336 patients included in the Dutch Lung Surgery Audit,^7^ showed that only 54% of patients were clinically staged accurately, and no comment could be made on whether this impacted on patient survival outcomes. Thus, to investigate further, we used individual participant data (IPD) from trials supplied for a systematic review and meta-analysis of preoperative chemotherapy in non-small cell lung cancer to assess the accuracy of clinical staging, factors that may affect inaccuracy, and how inaccuracy might impact on treatment decisions and survival.

## Methods

To be eligible for inclusion in the original IPD meta-analysis,^8^ trials should have randomized patients with NSCLC to preoperative chemotherapy followed by surgery (± postoperative radiotherapy) vs surgery (± postoperative radiotherapy). Full details of the methods are presented elsewhere.^8^ IPD were collected for 15 eligible randomized controlled trials and included 2,385 patients with non-small cell lung cancer.^8^ However, only data from patients from the control arm in these trials were used in this analysis, to ensure that any difference between clinical and pathologic staging could not have been influenced by preoperative chemotherapy. Included randomized controlled trials (RCTs) used different editions of TNM staging, and these changes over time were taken into account (e-Table 1).

Data on age, sex, clinical staging techniques, clinical TNM stage, extent of resection, pathologic TNM stage, histology, performance status, treatment group and dates of randomization, last follow-up, and death were collected. We approached study investigators for permission to use these data for these analyses and for clarification where staging methods were unclear in the original trial protocol or manuscript.

### Statistical Analysis

To assess agreement between clinical TNM stage (cTNM) and pathologic TNM stage (pTNM), a simple percentage agreement was calculated. Agreement between clinical and pathologic stage was also calculated using a weighted Cohen’s κ, which takes into account both agreement by chance and the degree of disagreement. κ statistics were categorized, as < 66% = low agreement, ≥ 66% = fair agreement, and ≥ 90% = good agreement.^9^, ^10^

To assess whether or not patient and trial characteristics might be associated with any cTNM staging inaccuracy age, sex, histology, year of randomization, and staging method were included in a multivariate logistic regression model. Histology was classified into adenocarcinoma, squamous, and other/unknown. Staging methods were classified as CT scan with or without a chest radiograph or CT scan plus any other staging method, as there were insufficient data to do this in more detail. Staging method correlated strongly with year of randomization, so we included only the former in our primary analysis. However, a sensitivity analysis was also performed, where staging method was replaced with year of randomization. We generated Kaplan-Meier curves for overall survival based on patients who were clinically understaged, clinically overstaged, and for those whose cTNM and pTNM agreed, and compared these using a log-rank test, stratified by trial and subsequently also pathologic stage. The accuracy of clinical T stage and nodal status were considered separately to help pinpoint which disagreements could have influenced treatment decisions.

## Results

Fifteen RCTs were included in the original IPD systematic review and meta-analysis of preoperative chemotherapy followed by surgery vs surgery alone. Nine trials^5^, ^11^, ^12^, ^13^, ^14^, ^15^, ^16^, ^17^, ^18^ (randomizing 1,586 patients in total) included data on both cTNM and pTNM stage, providing 698 control-arm patients for analysis (Table 1). These RCTs accrued patients between 1987 and 2005.

**Table 1:** Characteristics of Included Trials

Trial	Total Patients Randomized	Patients Randomized to Control Arm	Patients Who Provided Clinical and Pathologic Data	Accrual Period	Staging System (TNM)^a^	Staging Method	Surgical Protocol
M.D. Anderson (USA); Roth et al^11^/1994	60	32	32	1987-1993	4	Chest radiography	One or more positive nodal stations allowed. Patients with left lung tumors and paratracheal lymph node metastases excluded
MIP-91 (France); Depierre et al^12^/2002 (12, 29)	355	176	170	1991-1997	4	Chest radiography, CT imaging	Mediastinal node dissection and node sampling were left to the discretion of the surgeon
Netherlands; Splinter et al^13^/2000	79	40	37	1991-1999	4	CT imaging and mediastinoscopy	Mediastinal lymph node exploration was encouraged: for right-sided lesions, this included 2R, 4R, 7, 8, 9. For left-sided lesions, this included 4L, 5, 6, 7, 8, 9
JCOG 9209 (Japan); Nagai et al^14^/2003	62	31	31	1993-1998	4	CT imaging	Surgery was either lobectomy, bilobectomy, or pneumonectomy along with systematic mediastinal lymph node dissection
Finland; Mattson et al^15^/2003	62	32	23	1995-1999	4	CT imaging	“Local surgery”
MRC LU22 (UK); Gilligan et al^5^/2007	519	261	194	1997-2005	5/6	Bronchoscopy, mediastinoscopy, and CT imaging, PET	At cervical mediastinoscopy, the following lymph node stations will, wherever possible, be sampled: 2R, 2L, 4R, 4L, 7
SWOG S9900 (USA); Pisters et al^16^/2010	354	174	170	1999-2004	5/6	Chest radiography and CT imaging	All accessible hilar (level 10) lymph nodes must be dissected …A complete mediastinal lymph node sampling should be performed…for right-sided lesions, this includes 2R, 4R, 7, 8, and 9. For left-sided lesions, this includes 4L, 5, 6, 7, 8, and 9
China; Wu et al^17^/2002	55	23	20	1999-2004	5/6	Chest radiography, CT imaging, bronchoscopy and abdominal ultrasound	Surgery consisted of radical lung resection and systematic mediastinal lymph node dissection
China; Yang et al^18^/2005	40	21	21	1999-2004	5/6	Chest radiography, CT imaging, bronchoscopy and abdominal ultrasound	Lobectomy or pneumonectomy with systematic lymph node dissection

a For details of TNM staging systems, see e-Figure.

Clinical staging protocols varied among the trials (Table 1). One trial^11^ (which recruited patients between 1987 and 1993) used chest radiography and mediastinoscopy only. More recent trials used CT scans and PET-CT imaging, but no trial utilized PET-CT scanning routinely, such that only 67 patients included in the analysis underwent PET-CT imaging. There was also variation among trials in the surgical methods used (Table 1).

Of the 698 patients included, 318 (46%) were cTNM stage I (83% of which were Ia), 160 (23%) were cTNM stage II (91% of which were IIa), and 218 (31%) were cTNM stage IIIa (Table 2). Only two patients were classed as cTNM stage IIIB, and were therefore not included in the regression or survival analyses. A more detailed breakdown is given in e-Figure 1.

**Table 2:** Agreement Between Clinical and Pathologic TNM Stage Data

TNM Stage	TNM Stage					Total
	pI	pII	pIIIa	pIIIb	pIV	
cI	177 (25.4%)	72 (10.3%)^a^	44 (6.3%)^a^	22 (3.2%)^a^	3 (0.4%)^a^	318 (45.6%)
cII	40 (5.7%)^b^	67 (9.6%)	32 (4.6%)^a^	16 (2.3%)^a^	5 (0.7%)^a^	160 (22.9%)
cIIIa	32 (4.6%)^b^	28 (4.0%)^b^	116 (16.6%)	30 (4.3%)^a^	12 (1.7%)^a^	218 (31.2%)
cIIIb	0^b^	0^b^	0^b^	2 (0.3%)	0^a^	2 (0.3%)
cIV	0^b^	0^b^	0^b^	0^b^	0	0
Total	249 (35.7%)	167 (23.9%)	192 (27.5%)	70 (10.0%)	20 (2.9%)	698 (100%)

a Clinically understaged.

b Clinically overstaged.

Agreement between cTNM and pTNM staging was low (52%; weighted Cohen’s κ = 0.35; 95% CI, 0.30-0.40) (Table 2). In 34% of cases, patients were clinically understaged, and in 14% of cases, patients were clinically overstaged (Table 2). In the main regression analysis, age (*P* = .12), sex (*P* = .62), histology (*P* = .82), and the staging method (*P* = .32) were not significantly associated with the accuracy of cTNM staging, and in a sensitivity analysis there was no association with year of randomization (*P* = .98; e-Table 2).

**Figure 1:**
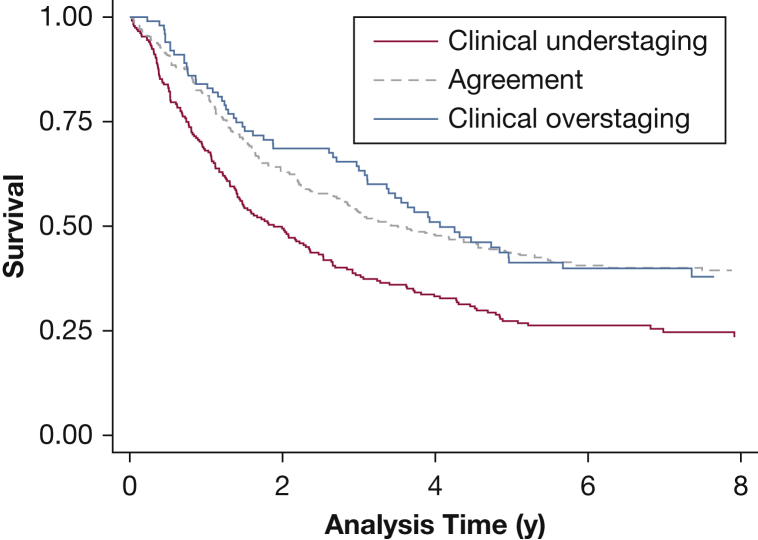
Kaplan-Meier curves for overall survival for all trial data combined, by agreement of clinical TNM staging with pathologic TNM staging.

**Figure 2:**
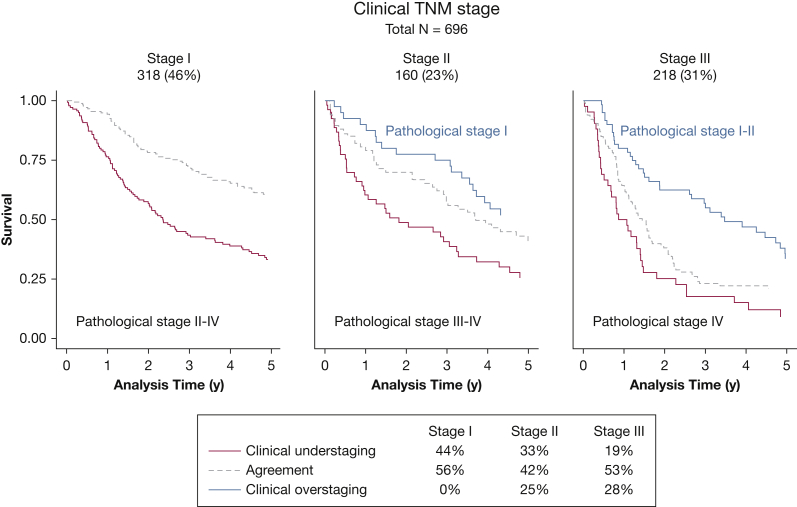
Kaplan-Meier curves for overall survival in clinically staged I, II, and III patients, by agreement of clinical TNM staging with pathologic TNM staging*.*

Survival varied with the accuracy of cTNM staging. In particular, patients who were clinically understaged appeared to have poorer survival than those who were clinically overstaged or those for whom cTNM and pTNM staging agreed (log-rank test stratified by trial *P* < .0001) (Fig 1). However, this is driven by the underlying pTNM stage (log-rank test stratified by trial and pathologic stage *P* = .54), which is more clearly illustrated in Figure 2. In particular, 44% of patients classed as cTNM stage I were pTNM stage II-IV, and 33% of patients classed as cTNM stage II were pTNM stage III-IV, explaining their lower survival (Fig 2).

Agreement was low between clinical and pathologic T stage (65%; weighted Cohen’s κ = 0.33; 95% CI, 0.27-0.39) (Table 3) and N stage (62%, weighted Cohen’s κ = 0.42; 95% CI, 0.37-0.48) (Table 4). Specifically, clinical staging failed to detect T4 disease in 10% of patients (Table 3), and nodal disease in 19% of patients. In addition, 12% were judged erroneously to have node-positive disease (Table 4).

**Table 3:** Agreement Between Clinical and Pathologic T Stage Data

T Stage	T Stage				Total
	pT1	pT2	pT3	pT4	
cT1	34 (4.9%)	16 (2.3%)^a^	3 (0.4%)^a^	7 (1.0%)^a^	60 (8.6%)
cT2	35 (5.0%)^b^	360 (51.6%)	69 (9.9%)^a^	40 (5.7%)^a^	504 (72.2%)
cT3	7 (1.0%)^b^	42 (6.0%)^b^	60 (8.6%)	23 (3.3%)^a^	132 (18.9%)
cT4	0^b^	0^b^	0^b^	2 (0.3%)	2 (0.3%)
Total	76 (10.9%)	418 (59.9%)	132 (18.9%)	72 (10.3%)	698 (100%)

a Clinically understaged.

b Clinically overstaged.

**Table 4:** Agreement Between Clinical and Pathologic Nodal Status Data

Nodal Status	Nodal Status				Total
	pN0	pN1	pN2	pN3	
cN0	259 (37.1%)	74 (10.6%)^a^	57 (8.2%)^a^	1 (0.1%)^a^	391 (56.0%)
cN1	56 (8.0%)^b^	67 (9.6%)	29 (4.2%)^a^	0^a^	152 (21.8%)
cN2	28 (4.0%)^b^	19 (2.7%)^b^	104 (14.9%)	4 (0.6%)^a^	155 (22.2%)
cN3	0^b^	0^b^	0^b^	0	0
Total	343 (49.1%)	160 (22.9%)	190 (27.2%)	5 (0.7%)	698 (100%)

a Clinically understaged.

b Clinically overstaged.

## Discussion

### Results Summary

We found that cTNM stage disagreed with pTNM stage in about one-half of patients, and was not clearly associated with age, sex, histology, the staging method used, or year of randomization. The discrepancies between clinical and pathologic T staging and N staging could have led to different treatment decisions in 10% and 38% of cases, respectively.

### Strengths

To our knowledge, this is the first time IPD from major RCTs have been combined to assess the accuracy of staging in stage I-III NSCLC. While the randomized controlled trials included did not intend to evaluate staging, with the agreement of those who provided the data, this novel methodology provided us with a valuable opportunity to investigate more reliably the accuracy of clinical TNM staging. We could take advantage of per-protocol clinical staging and surgery and rigorous documentation of clinical and pathologic TNM stage for each patient. Also, data from randomized trials are less susceptible to the selection biases that can affect cohort studies.^19^, ^20^ Using IPD has enabled us to restrict the analysis to the control arms of these trials, thus avoiding confounding by treatment received and, in particular, potential downstaging from use of preoperative chemotherapy.

For the first time, to our knowledge, this study also demonstrates the impact of the inaccuracy of clinical staging on patient survival outcomes. Importantly, the impact of staging accuracy on clinical decision making is also demonstrated using unselected data. The poorer survival seen in clinically understaged patients was explained by the underlying pTNM stage.

### Limitations

Over time the trials included here used increasingly sophisticated staging methods, but surprisingly, a significant improvement in accuracy was not seen. However, many of the staging methods utilized in the included trials may now be considered suboptimal.^21^ Earlier studies employed CT scanning and mediastinoscopy while the most recent trial used additional PET-CT imaging, but none used endosonography. Despite this, our staging accuracy results are remarkably similar to those from the audit of the quality of staging in Dutch patients,^7^ which included routine use of PET-CT imaging and endosonography and included patients from January 2013 to December 2014. Indeed, of the patients included in our analysis that did undergo PET-CT imaging, one-quarter of patients were still understaged and this is discussed elsewhere.^6^ While PET-CT imaging or endosonography was not routinely utilized in the trials included in this meta-analysis, this practice reflects current American College of Chest Physicians guidance^22^ for patients with stage IA disease, which does not recommend the use of PET imaging or endosonography. Although it is difficult to generalize, assuming the trial population reflects routine practice, the data here suggest that 44% of patients with clinical stage I disease might have more advanced disease diagnosed postoperatively. A further limitation is that intraoperative pathologic staging protocols may have varied and are unlikely to be as comprehensive as currently recommended.^23^ However, incomplete pathologic staging would only serve to reduce the extent of nodal staging inaccuracy.

### Context

The advent of stereotactic radiotherapy and radiofrequency ablation for the treatment of early-stage NSCLC has highlighted the importance of accurate nodal staging. These newer techniques are used for the treatment of early-stage lung cancer but, in contrast to surgery, do not provide pathologic staging information. In a study of relapse of NSCLC following stereotactic radiotherapy or surgery, there were twice as many recurrences in local lymph nodes in patients undergoing stereotactic radiotherapy compared with surgery,^24^ emphasizing the importance of accurate nodal staging prior to SABR.

When surgery is undertaken and pathologic staging is available, prior invasive mediastinal sampling may take on less significance if we assume that surgery followed by adjuvant chemotherapy is at least as effective as chemoradiation. When considering stage II and III disease, inaccurate clinical staging may reduce the efficacy of surgery by failing to detect multistation N2 or N3 disease. For patients undergoing radical radiotherapy, imprecise clinical staging can result in an incorrect radiation field.

The most likely explanation for the low level of accuracy of clinical staging for patients with operable NSCLC is the sensitivity of the diagnostic tools employed. Patients being considered for treatment with curative intent typically undergo CT and PET-CT imaging as well as mediastinal sampling when required. Using a 10-mm short-axis cutoff for significance of mediastinal nodes, the sensitivity of CT scanning in detecting mediastinal metastases is 55%.^22^ PET-CT imaging has a sensitivity of 77% to 81%^25^ and may vary according to brand of scanner and histology. In a systematic pooled analysis of 9,267 patients, mediastinoscopy had a sensitivity of 78%.^22^ Overstaging may occur with PET-CT imaging unless current guidelines^22^ are adhered to and PET-positive findings are clarified by invasive sampling. More recently the introduction of endobronchial and endoscopic ultrasound has improved the clinical staging of patients with NSCLC, resulting in a reduction in futile surgery^26^, ^27^ and potentially increased survival^28^ when employed routinely for patients with stage I-III disease.

### Implications

These findings have implications for the care of patients with NSCLC, as well as appropriate selection of suitable patients for inclusion in clinical trials. Understaging the T stage may mean that the patient undergoes surgery without the surgeon knowing the full extent of the primary disease, which may result in an incomplete resection. Ten percent of patients in our analysis were found to have previously unexpected T4 disease. Erroneous nodal staging in patients without metastatic disease can similarly result in inappropriate treatment decisions, which can significantly impact on patient outcomes. Patients with nodal disease undetected by clinical staging methods may undergo futile surgery (or SABR) whereas chemoradiotherapy may have been the preferred initial treatment of clinicians and patients with full knowledge of nodal involvement. Conversely, if clinical staging overestimates the extent of nodal disease (114 patients [15%] in this meta-analysis), then this may mean patients are denied potentially curative surgery. The data for this analysis were obtained from patients in controlled clinical trials, generally from centers with lung cancer expertise. Therefore, clinical staging accuracy in the wider population could well be worse.

## Conclusions

The results of this analysis highlight some flaws in the clinical care of patients with NSCLC and emphasize the need for further research into techniques for improving staging accuracy for patients with stage I-III NSCLC.

## Supplementary Data

e-Online Data

Audio

## References

[1] Brierley J.D., Gospodarowicz M.K., Wittekind C. (2016). TNM Classification of Malignant Tumours.

[2] Timmerman R., Paulus R., Galvin J. (2010). Stereotactic body radiation therapy for inoperable early stage lung cancer. JAMA.

[3] Lencioni R., Crocetti L., Cioni R. (2008). Response to radiofrequency ablation of pulmonary tumours: a prospective, intention-to-treat, multicentre clinical trial (the RAPTURE study). Lancet Oncol.

[4] Cerfolio R.J., Bryant A.S., Ojha B., Eloubeidi M. (2005). Improving the inaccuracies of clinical staging of patients with NSCLC: a prospective trial. Ann Thorac Surg.

[5] Gilligan D., Nicolson M., Smith I. (2007). Preoperative chemotherapy in patients with resectable non-small cell lung cancer: results of the MRC LU22/NVALT2/EORTC 08012 multicentre randomised trial and update of systematic review. Lancet.

[6] Navani N., Nankivell M., Stephens R.J. (2010). Inaccurate clinical nodal staging of non-small cell lung cancer: evidence from the MRC LU22 multicentre randomised trial. Thorax.

[7] Heineman D.J., Geert ten Berge M., Daniels J.M. (2016). The quality of staging non-small cell lung cancer in the Netherlands: data from the Dutch Lung Surgery Audit. Ann Thorac Surg.

[8] NSCLC Meta-analysis Collaborative Group (2014). Preoperative chemotherapy for non-small cell lung cancer: a systematic review and meta-analysis of individual participant data. Lancet.

[9] McMinn A.M., van Sluijs E.M.F., Harvey N.C. (2009). Validation of a maternal questionnaire on correlates of physical activity in preschool children. Int J Behav Nutr Phys Act.

[10] Portney L.G., Watkins M.P. (2000). Foundations of Clinical Research: Applications to Practice.

[11] Roth J.A., Fosella F., Komaki R. (1994). A randomized trial comparing perioperative chemotherapy and surgery with surgery alone in resectable stage IIIa non-small cell lung cancer. J Natl Cancer Inst.

[12] Depierre A., Milleron B., Moro-Sibilot D. (2002). Preoperative chemotherapy followed by surgery compared with primary surgery in resectable stage I (except T1N0), II and IIIa non-small cell lung cancer. J Clin Oncol.

[13] Splinter T.A., van Putten J.W., Meuzalaar J., Smit E.F., Kho G.S., Groen H.J. (2000). Randomized multicentre phase II study of chemotherapy followed by surgery versus surgery alone in stage I and II non-small cell lung cancer (NSCLC). Proc Am Soc Clin Oncol.

[14] Nagai K., Tsuchiya R., Mori T. (2003). A randomised trial comparing induction chemotherapy followed by surgery with surgery alone for patients with stage IIIa N2 non-small cell lung cancer. J Thorac Cardiovasc Surg.

[15] Mattson K.V., Abratt R.P., ten Velde G., Krofta K. (2003). Docetaxel as neoadjuvant therapy for radically treatable stage III non-small cell lung cancer: a multinational randomised phase III study. Ann Oncol.

[16] Pisters K.M., Vallieres E., Crowley J.J. (2010). Surgery with or without preoperative paclitaxel and carboplatin in early-stage non-small-cell lung cancer: Southwest Oncology Group Trial S9900, an intergroup, randomized, phase III trial. J Clin Oncol.

[17] Wu Y.-L., Gu L.-J., Weng Y.-M., Feng W.-N., Cheng C. (2002). Neo-adjuvant chemotherapy with docetaxel plus carboplatin for non-small cell lung cancer. Ann Oncol.

[18] Yang X., Wu Y., Gu L. (2005). A randomized trial comparing neoadjuvant gemcitabine plus carboplatin or cisplatin followed by surgery with surgery alone in clinical stage IIIA non-small-cell lung cancer (NSCLC). Lung Cancer.

[19] Lopez-Encuentra A., Garcia-Lujan R., Rivas J.J., Rodriguez-Rodriguez J., Torres-Lanza J., Varela-Simo G. (2005). Comparison between clinical and pathologic staging in 2994 cases of lung cancer. Ann Thorac Surg.

[20] Stiles B.M., Servais E.L., Lee P.C., Port J.L., Paul S., Altorki N.K. (2009). Point: Clinical stage IA non-small cell lung cancer determined by computed tomography and positron emission tomography is frequently not pathologic IA non-small cell lung cancer: the problem of understaging. J Thorac Cardiovasc Surg.

[21] Postmus P.E., Kerr K.M., Oudkerk M. (2017). Early and locally advanced non-small cell lung cancer (NSCLC): ESMO clinical practice guidelines for diagnosis, treatment and follow-up. Ann Oncol.

[22] Silvestri G.A., Gonzalez A.V., Jantz M.A. (2013). Methods for staging non-small cell lung cancer: diagnosis and management of lung cancer, 3rd ed: American College of Chest Physicians evidence-based clinical practice guidelines. Chest.

[23] Lardinois D., De Leyn P., Van Schil P. (2006). ESTS guidelines for intraoperative lymph node staging in non-small cell lung cancer. Eur J Cardiothorac Surg.

[24] van den Berg L.L., Klinkenberg T.J., Groen H.J., Widder J. (2015). Patterns of recurrence and survival after surgery or stereotactic radiotherapy for early stage NSCLC. J Thorac Oncol.

[25] Schmidt-Hansen M., Baldwin D.R., Zamora J. (2015). FDG-PET/CT imaging for mediastinal staging in patients with potentially resectable non-small cell lung cancer. JAMA.

[26] Annema J.T., van Meerbeeck J.P., Rintoul R.C. (2010). Mediastinoscopy vs endosonography for mediastinal nodal staging of lung cancer: a randomized trial. JAMA.

[27] Korevaar D.A., Crombag L.M., Cohen J.F., Spijker R., Bossuyt P.M., Annema J.T. (2016). Added value of combined endobronchial and oesophageal endosonography for mediastinal nodal staging in lung cancer: a systematic review and meta-analysis. Lancet Respir Med.

[28] Navani N., Nankivell M., Lawrence D.R. (2015). Lung cancer diagnosis and staging with endobronchial ultrasound-guided transbronchial needle aspiration compared with conventional approaches: an open-label, pragmatic, randomised controlled trial. Lancet Respir Med.

