# Supplementary material for: The Accuracy of Clinical Staging of Stage I-IIIa Non-Small Cell Lung Cancer: An Analysis Based on Individual Participant Data
Source: Chest. 2018 Oct 26;155(3):502–9. doi: 10.1016/j.chest.2018.10.020 (PMC6435782; doi:10.1016/j.chest.2018.10.020)
Supplement: e-Online Data [file mmc1.pdf]

CHEST Online Supplement

The Accuracy of Clinical Staging of Stage I-IIIa Non-Small Cell Lung Cancer

An Analysis Based on Individual Participant Data

Neal Navani, MD, PhD; David J. Fisher, MSc; Jayne F. Tierney, PhD; Richard J. Stephens; and Sarah Burdett, MSc; on behalf of the NSCLC Meta-analysis Collaborative Group

CHEST 2019; 155(3):502-509

Online supplements are not copyedited prior to posting and the author(s) take full responsibility for the accuracy of all data.

© 2018 AMERICAN COLLEGE OF CHEST PHYSICIANS. Reproduction of this article is prohibited without written permission from the American College of Chest Physicians. See online for more details. **DOI:** 10.1016/j.chest.2018.10.020

e-Table 1.

Comparison of TNM staging systems

4th edition (1987)(29)		5th edition (1997)(30), 6th edition (2002)(31)	
Stage I	T1,N0,M0<br>T2,N0,M0	Stage IA<br>Stage IB	T1,N0,M0<br>T2,N0,M0
Stage II	T1,N1,M0<br>T2,N1,M0	Stage IIA<br>Stage IIB	T1,N1,M0<br>T2,N1,M0<br>T3,N0,M0
Stage IIIA<br><br><br>Stage IIIB	T1,N2,M0<br>T2,N2,M0<br>T3,N0/1/2,M0<br>anyT,N3,M0<br>T4,anyN,M0	Stage IIIA<br><br><br>Stage IIIB	T1,N2,M0<br>T2,N2,M0<br>T3,N1/2,M0<br>anyT,N3,M0<br>T4,anyN,M0
Stage IV	anyT, anyN, M1	Stage IV	anyT,anyN,m1

Online supplements are not copyedited prior to posting and the author(s) take full responsibility for the accuracy of all data.

e-Figure 1. Flowchart describing clinical and pathological agreement, clinical over staging and clinical under staging

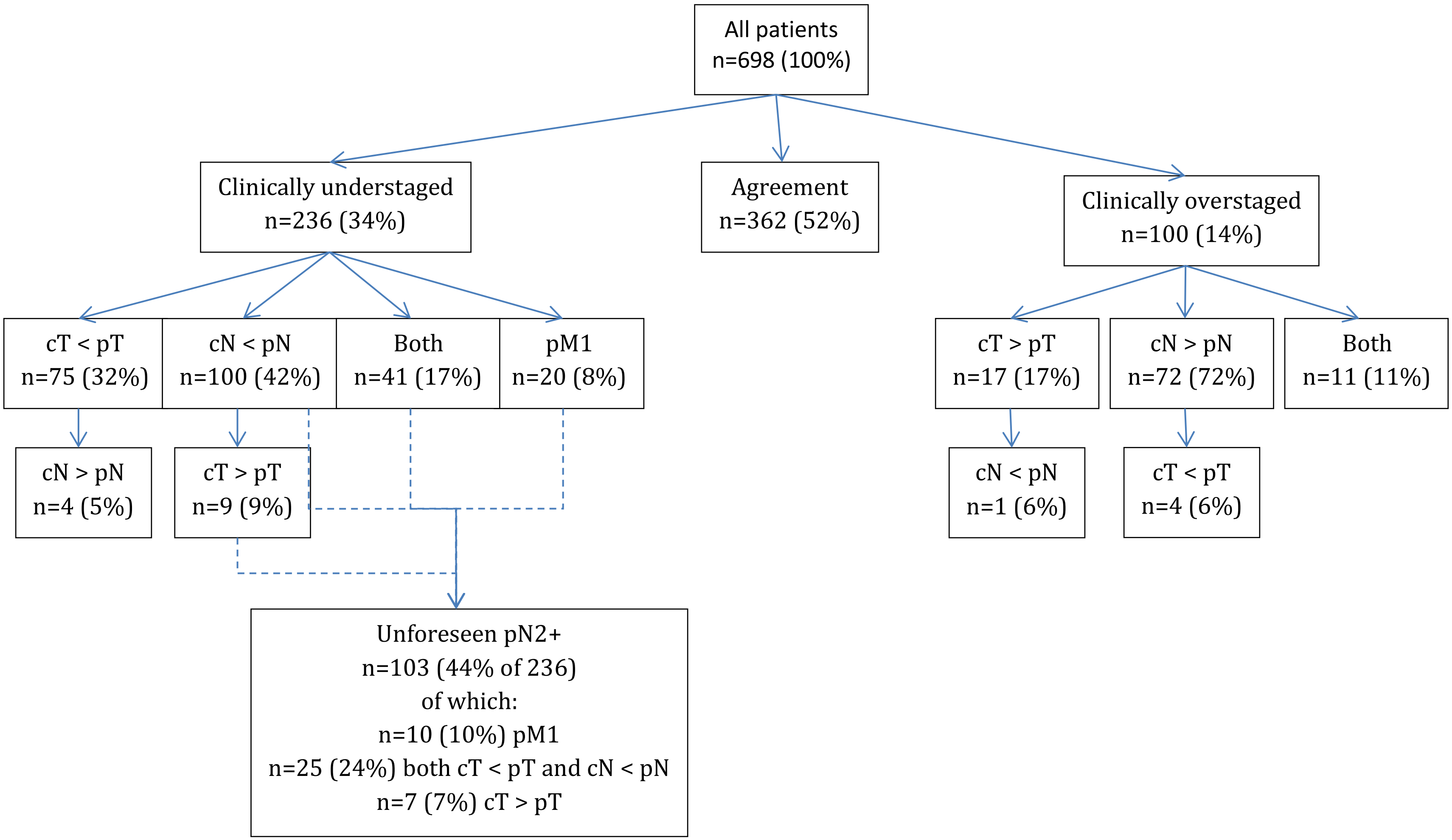

Online supplements are not copyedited prior to posting and the author(s) take full responsibility for the accuracy of all data.

e-Table 2.

Multivariate logistic regression; Factors that may predict staging agreement

**Predictor**	**TNM stage**	
	**χ^2 (df)**	**p-value**
Histology	0.40 (2)	0.82
Staging method	1.01 (1)	0.32
Age	2.48 (1)	0.12
Gender	0.24 (1)	0.62
**Overall***	**4.22 (5)**	**0.52**

"Overall" compares the model with all covariates entered to the null model

Sensitivity analysis with staging method replaced with year of accrual:

**Predictor**	**TNM stage**	
	**χ^2 (df)**	**p-value**
Histology	0.48 (2)	0.79
Year of randomisation	0.00 (1)	0.98
Age	2.55 (1)	0.11
Gender	0.19 (1)	0.66
**Overall***	**3.21 (5)**	**0.67**

Online supplements are not copyedited prior to posting and the author(s) take full responsibility for the accuracy of all data.
